# Application of EUS or MRCP prior to ERCP in patients with suspected choledocholithiasis in clinical practice

**DOI:** 10.1055/a-2475-0099

**Published:** 2025-01-07

**Authors:** Mike J.P. de Jong, Megan M.L. Engels, Christa Sperna Weiland, Robin Krol, Tanya M. Bisseling, Erwin-Jan M. van Geenen, Peter Siersema, Foke van Delft, Jeanin E. van Hooft

**Affiliations:** 1 6034 Gastroenterology and Hepatology, Radboud University Medical Center, Nijmegen, Netherlands; 2 6028 Research and Development, St Antonius Hospital, Nieuwegein, Netherlands; 3 4501 Gastroenterology and Hepatology, Leiden University Medical Center, Leiden, Netherlands; 4 10233 Gastroenterology and Hepatology, Jeroen Bosch Hospital, 's-Hertogenbosch, Netherlands; 5 72489 Gastroenterology and Hepatology, Maas Hospital Pantein, Boxmeer, Netherlands; 6 6993 Gastroenterology and Hepatology, Erasmus Medical Center, Rotterdam, Netherlands

**Keywords:** Pancreatobiliary (ERCP/PTCD), Stones, Endoscopic ultrasonography, Biliary tract, Quality and logistical aspects, Performance and complications, ERC topics

## Abstract

**Background and study aims** Patients with symptomatic cholelithiasis can be stratified according to the 2019 European Society for Gastrointestinal Endoscopy (ESGE) guideline into low-, intermediate- and high-likelihood groups for presence of choledocholithiasis. For the intermediate group, endoscopic ultrasound (EUS) or magnetic resonance cholangiopancreatography (MRCP) is recommended to assess whether an endoscopic retrograde cholangiopancreatography (ERCP) is necessary prior to cholecystectomy. The aim of the study was to investigate adherence to the guideline for diagnostic and treatment strategy for cholelithiasis in daily clinical practice.

**Patients and methods** A multicenter, retrospective cross-sectional observational study of the diagnostic pathway of patients with suspicion of choledocholithiasis was conducted between 2019 and 2021. Patients were stratified according to the ESGE guideline "Endoscopic management of common bile duct stones”.

**Results** A total of 305 patients were included in the analysis and stratified into low- (17%), intermediate- (40%) and high- (43%) likelihood of choledocholithiasis. In these three categories, 182 patients (60%) underwent ERCP. Adherence to the ESGE guideline recommendation was 59.7% overall and was the highest in the intermediate-likelihood group (83.6%), compared with 45.1% in the low- and 43.2% in the high-likelihood group, respectively ( *P* < 0.001). In the high-likelihood group, 49% underwent additional imaging. In 195 patients who underwent additional imaging, 55 ERCPs (28.2%) could be avoided.

**Conclusions** This study shows that stratification according to the ESGE guideline is useful to reduce the number of unnecessary additional imaging procedures and ERCPs in patients with a suspicion of choledocholithiasis. It seems worthwhile to perform EUS prior to ERCP in the same session.

## Introduction

Cholelithiasis is frequently encountered in the general population, with a prevalence of 20% 1 . In 10% to 15% of patients, symptoms are caused by an impacted stone in the common bile duct (CBD) 2 . Given the risk of adverse events (AEs), such as cholangitis, pancreatitis 3 or post-cholecystectomy bile leakage 4 , patients with choledocholithiasis require endoscopic retrograde cholangiopancreatography (ERCP) to remove bile duct stones in the CBD prior to cholecystectomy 5 .

ERCP is associated with a considerable incidence of complications, with post-ERCP pancreatitis (PEP) being the most prevalent, exhibiting an incidence of 10.2% with a mortality rate of 0.2% 6 . Therefore, only patients who have been definitively diagnosed with choledocholithiasis should undergo ERCP. To determine the appropriate treatment course, patients with symptomatic cholelithiasis can be stratified according to choledocholithiasis likelihood by either the 2019 American Society for Gastrointestinal Endoscopy (ASGE) or European Societies of Gastrointestinal Endoscopy (ESGE) guidelines 5 7 .

The initial diagnostic workup consists of bilirubin and liver function tests (LFTs) in blood and abdominal ultrasound (US). If both are unremarkable, the likelihood of choledocholithiasis is estimated to be less than 10% (low likelihood) and patients can proceed directly to cholecystectomy without the need for additional imaging. Conversely, if choledocholithiasis is detected on abdominal US or cholangitis is present, the ESGE classifies patients as high likelihood (> 50% risk of choledocholithiasis) and they can proceed directly to ERCP without additional imaging ( **Supplementary material 1** ) 5 .

Additional imaging, with endoscopic US (EUS) or magnetic resonance cholangiopancreatography (MRCP), is indicated for patients in the intermediate-likelihood group (who have a calculated risk of 10% to 50% of choledocholithiasis) to reduce risk of undetected choledocholithiasis during cholecystectomy or a futile ERCP procedure. This intermediate-likelihood group consists of patients with abnormal LFTs and/or dilated CBD on US and poses a treatment dilemma, given the lack of visible choledocholithiasis or clinical cholangitis 5 .

Although a randomized controlled trial comparing EUS with MRCP in patients with intermediate likelihood showed no significant difference in detecting choledocholithiasis 8 , a previously conducted meta-analysis demonstrated better pooled sensitivity and specificity (97% and 90%, respectively) for EUS in smaller and less rigorously selected populations 9 . It has been hypothesized that performing EUS in combination with ERCP can offer many benefits: reduced hospital stay length, decreased sedation procedures, and prevention of interim adverse biliary events 10 . Furthermore, performing a diagnostic EUS, with an overall complication rate of 0.034%, can prevent an unnecessarily performed ERCP associated with a 12% complication risk 11 . Moreover, same-session EUS allows the endoscopist to examine luminal and duct anatomy, which can be helpful for successfully performing ERCP. Only a few studies have addressed timing of additional imaging prior to ERCP and the potential advantage of same-procedure EUS as opposed to MRCP in preventing treatment delay 12 .

The primary aim of this study was to assess how additional diagnostic imaging (EUS or MRCP) is applied in combination with ERCP for patients with suspected choledocholithiasis since publication of the 2019 ESGE guideline. Second, this study examined possible improvement measures for preventing AEs, treatment delay, and unnecessarily performed therapeutic ERCPs.

## Patients and methods

### Study design

This study was designed as a multicenter, cross-sectional, observational cohort study. It was conducted according to the principles of the Declaration of Helsinki 13 and in accordance with the Medical Research Involving Human Subjects Act. A Strengthening the Reporting of Observational studies in Epidemiology (STROBE) checklist is included in the supplements ( **Supplementary material 3** ) 14 . The study was approved by the medical ethical committee (#2022–15783) of the Radboud university medical center, Nijmegen, The Netherlands. Written informed consent was waived for Radboud university medical center, Nijmegen patients who were treated in the institution. For patients from Maasziekenhuis Pantein, written informed consent was obtained.

### Participants

This study investigated implementation of the 2019 ESGE guideline in clinical practice. Therefore, the years 2019 to 2021 were chosen because they followed the most recent guideline publication 5 . Sample size was determined by the available data. Patients with suspected choledocholithiasis or cholangitis who presented to Radboud university medical center, Nijmegen (tertiary center) or Maasziekenhuis Pantein, Beugen, The Netherlands (community hospital) between 2019 and 2021 were retrospectively reviewed. To be eligible to participate in this study, the following inclusion criteria applied: age ≥ 18 years and suspicion of choledocholithiasis during initial diagnostic workup (including LFTs and/or abdominal US). Patients with concurrent pancreaticobiliary malignancy or an indication other than choledocholithiasis or cholangitis for additional imaging/ERCP were excluded. Also, patients with a non-gallstone-related etiology of cholangitis (e.g., stricturing biliary disease or stent dysfunction) were excluded.

### Study procedures and data collection

Data were extracted from the electronic patient record systems of both Radboud university medical center and Maasziekenhuis Pantein for all patients with a billing code of choledocholithiasis or extrahepatic bile duct pathology in the years 2019, 2020, and 2021. After screening for inclusion and exclusion criteria, each patient was stratified into the low-, intermediate-, or high-likelihood category of choledocholithiasis according to the 2019 ESGE guideline based on standard diagnostic workup. The electronic patient record system was used to collect the necessary data in the data management system CastorEDC (Castor Electronic Data Capture, Amsterdam, the Netherlands: Ciwit BV 2019). Clinical data about patient demographics, diagnostic workup, eventual treatment strategy, and gallstone- or treatment-related complications during available follow-up were collected.

If a computed tomography (CT) scan had been performed instead of an abdominal US, this CT scan was considered as first imaging modality, and the diagnostic workup was classified as adequate. EUS and/or MRCP were not considered appropriate first imaging modalities. Abnormal LFTs were counted as bilirubin or alkaline phosphatase above the upper limit of normal (ULN). The number of patients with 2x > ULN were also listed in the baseline table because this value has been taken as cut-off in other related studies 15 . If either LFTs or US/CT were missing, patients were stratified solely according to the available diagnostic information.

Cholangitis was scored according to the Tokyo guideline 16 . Accordingly, the definition of suspected cholangitis was systemic inflammation combined with either cholestasis (jaundice or abnormal LFTs) or suspected abdominal US imaging (biliary dilatation or evidence of the etiology on imaging). Definitive diagnosis was only selected when all three criteria were present: systemic inflammation in combination with both cholestasis and suspected imaging. Both suspected and definitive cholangitis were classified as high likelihood for presence of choledocholithiasis.

### Endpoints

The primary endpoint was adherence to the ESGE guideline regarding additional imaging in patients with suspected choledocholithiasis. We evaluated whether stratification was performed according to guideline recommendations and whether the appropriate intervention was allocated after stratification (laparoscopic cholecystectomy, EUS/MRCP or ERCP). Adherence was defined as performing additional imaging in the intermediate group and not performing it in the low- and high-likelihood groups.

Second, we analyzed how many ERCPs were still performed after adequate negative workup in patients in the low- and intermediate-likelihood groups and how many futile ERCPs were performed in the high-likelihood group when additional imaging was not performed. In addition, we calculated the time interval between EUS/MRCP and ERCP.

Intraprocedural complications were defined as bleeding or perforation recognized and treated during the procedure. Postprocedural complications for ERCP or EUS were defined as follows: 1) PEP according to the Atlanta criteria 17 ; 2) cholangitis according to Tokyo guideline 16 ; 3) bleeding as a hemoglobin decrease of > 2 mmol/L within 48 hours post-procedure; 4) perforation as an iatrogenic transmural duodenal, stomach, or CBD tear requiring surgical, radiologic or endoscopic intervention or prolonged admission 18 . Severity of complications was scored according to Clavien-Dindo classification 19 .

### Statistical analysis

After final data collection, the database was locked and exported to IBM SPSS Statistics for Windows, version 27, for statistical analysis. Statistical analysis was performed by the study coordinator with the help of an independent statistician. Categorical data were summarized in count and percentages. Continuous data were summarized by either mean and standard deviation (SD) or median and interquartile range (IQR), depending on whether the data follow a normal distribution. *P* < 0.05 was considered statistically significant. All results are presented in a descriptive manner.

## Results

**Fig. 1:**
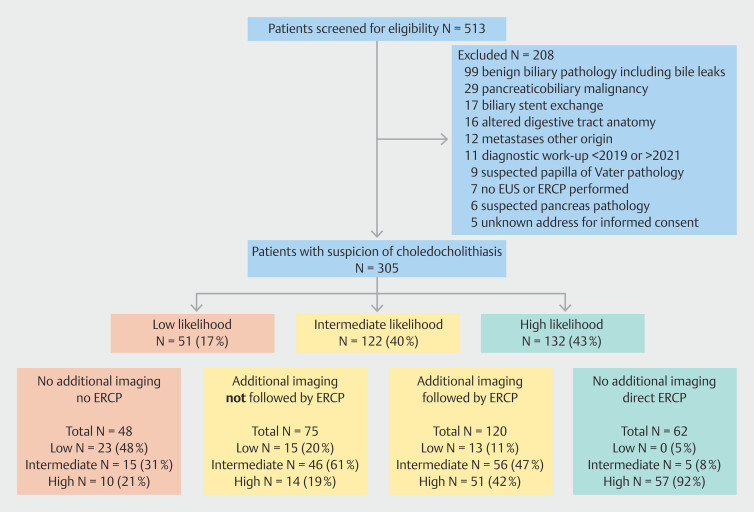
Inclusion and exclusion, likelihood groups, and treatment allocation. EUS, endoscopic ultrasound; ERCP, endoscopic retrograde cholangiopancreatography.

Between 2019–2021, 513 patients had a diagnostic workup with billing code choledocholithiasis or extrahepatic bile duct pathology. Of these patients, 208 (40.5%) were excluded, predominantly due to malignant biliary obstruction or benign obstruction, other than choledocholithiasis. This resulted in a final cohort of 305 patients ( Fig. 1 ). After stratification according to the ESGE guideline, 51 patients (17%) fell into the low-likelihood group, 122 (40%) in the intermediate-likelihood group, and 132 (43%) patients were stratified as high likelihood for having choledocholithiasis. ( Fig. 1 ).

### Baseline characteristics

Median age of the 305 included patients was 69 years (IQR 53–77 years). Of the patients, 119 (39.0%) were male and the median body mass index (BMI) was 26.2 (IQR 23.6–30.2) kg/m ^2^ . Patient age after likelihood stratification differed among groups, with the low-likelihood group comprising the youngest patients and the high-likelihood group the oldest. The exact difference is shown in Table 1 . Prior to diagnostic workup, 77 patients had a history of cholecystectomy, which was proportionally more frequent in the low-likelihood group compared with the intermediate- and high-likelihood groups (39.2% vs. 25.4% and 19.7%, respectively).

**Table:** **Table 1** Baseline characteristics and diagnostic workup.

	**Total (N = 305)**	**Low (N = 51)**	**Intermediate (N = 122)**	**High (N = 132)**
Age at EUS/ERCP – median (IQR)	69 (53–77)	51 (37–70)	66 (53–76)	74 (63–82)
Male - %	119 (39.0)	13 (25.5)	47 (38.5)	59 (44.7)
BMI – median (IQR) (N=286)	26.2 (23.6–30.2)	26.1 (22.7–30.9)	27.2 (24.4–30.5)	25.6 (23.1–29.3)
Pre-workup cholecystectomy - %	77 (25.2)	20 (39.2)	31 (25.4)	26 (19.7)
Complete diagnostic workup - %	261 (85.6)	36 (70.6)	112 (91.8)	113 (85.6)
Cholangitis - %				
No	213 (69.8)	51 (100%)	122 (100)	40 (30.3)
Suspected	41 (13.4)	0 (0)	0 (0)	41 (31.1)
Definitive	51 (16.7)	0 (0)	0 (0)	51 (38.6)
LFTs (N = 297) - %				
Normal	94 (31.6)	42 (89.4)	33 (27.3)	19 (14.7)
ULN	48 (16.2)	4 (8.5)*	28 (23.1)	16 (12.4)
2xULN	155 (52.2)	1 (2.1)*	60 (49.6)	94 (72.9)
Transabdominal US or CT - %				
No abnormalities	128 (42.0)	39 (76.5)	53 (43.4)	36 (27.3)
Dilated CBD	74 (24.3)	0 (0)	51 (41.8)	23 (17.4)
Impacted CBD stone	56 (18.4)	0 (0)	0 (0.0)	56 (42.4)
Other findings	8 (2.6)	0 (0)	8 (6.6)	0 (0)
Both not performed	39 (12.8)	12 (23.5)	10 (8.2)	17 (12.9)
*Persistent elevation of LFTs by steatosis, autoimmune hepatitis or other chronic liver disease. BMI, body mass index; CBD, common bile duct; CT, computed tomography; ERCP, endoscopic retrograde cholangiopancreatography; EUS, endoscopic ultrasound; LFT, liver function test; ULN, upper limit of normal; US, (abdominal) ultrasound.				

### Adherence to ESGE guideline

As shown in Table 2 , 182 of 305 included patients (59.7%) underwent the recommended diagnostic and treatment strategy according to the ESGE guideline. Adherence was best in the intermediate-likelihood group, at 83.6% compared with 45.1% and 43.2% in the low- and high-likelihood groups, respectively ( *P* < 0.001). Given that sex, BMI, and prior cholecystectomy could affect the a priori chance of CBD stones and subsequent deviation from the guideline, additional analyzes for these variables were performed. No statistically significant difference in guideline adherence for these variables was found.

**Table:** **Table 2** Primary outcome: adherence to ESGE recommendation for additional imaging.

	**Adherence to additional ESGE-recommended imaging**		
	**Yes**	**No**	***P* value **
**Total adherence**	182 (59.7)	123 (40.3)	
**Primary outcome measure**			
Likelihood groups			< 0.001
Low (N = 51)	23 (45.1)	28 (54.9)	
Intermediate (N = 122)	102 (83.6)	20 (16.4)	
High (N = 132)	57 (43.2)	75 (56.8)	
**Potential confounders**			
Sex			0.476
Female	114 (61.3)	72 (38.7)	
Male	68 (57.1)	51 (42.9)	
BMI			0.661
< 25	69 (62.7)	41 (37.3)	
25–30	60 (58.3)	43 (41.7)	
> 30	41 (56.2)	32 (43.8)	
Prior cholecystectomy			0.433
Yes	45 (58.4)	32 (41.6)	
No	63 (64.9)	34 (35.1)	
BMI, body mass index; ESGE, European Society for Gastrointestinal Endoscopy.			

### Diagnostic workup by LFTs and US

The initial diagnostic workup composed of LFTs and/or US. LFTs fell outside the normal range in 203 of 297 patients (68.4%) and were above 2x ULN in 155 of 297 patients (52.2%), with data missing for eight patients ( **Supplemental material 2** ). Of all patients, 266 (87.2%) underwent an abdominal US or CT scan in the initial workup, with no abnormalities in 42% of the imaging procedures. An impacted CBD stone was found in 18.4% of patients. All together, a complete initial diagnostic workup was performed in 261 of 297 patients (85.6%). After initial diagnostic workup, no additional imaging or ERCP was performed in 48 patients (23 patients in the low-likelihood group, 15 in the intermediate-likelihood group, and 10 in the high-likelihood group). Of the 10 patients in the high-likelihood group who underwent neither additional imaging nor ERCP, six were terminally ill and died shortly after diagnostic workup. The other four showed improvement in LFTs, suggesting spontaneous stone passage.

### Additional imaging

**Fig. 2:**
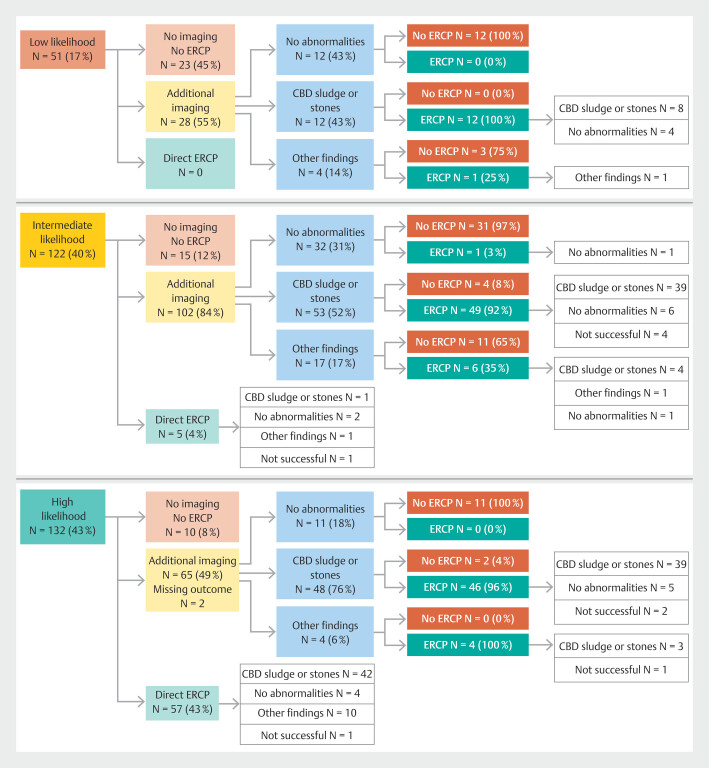
Likelihood groups with additional imaging + ERCP results. CBD, common bile duct; ERCP, endoscopic retrograde cholangiopancreatography.

In patients who underwent additional imaging (n = 195), the median interval between EUS and ERCP was 0 days (IQR 0–0); the median interval between MRCP and ERCP was longer, up to 10 days (IQR 3–37). CBD stones and/or sludge were found in 58.5% of the performed additional imaging. MRCP alone was the most used modality (42.1%) ( Table 3 ). For the low- and high-likelihood groups, 54.9% and 49.2% of patients (28 of 51 and 65 of 132, respectively) underwent additional imaging not recommended by the guideline ( Fig. 1 ). In total, performing additional imaging in 195 patients resulted in prevention of 55 ERCPs ( Fig. 2 ). In the high-likelihood group, no abnormalities were detected on additional imaging in 11 of 65 instances (16.9%), resulting in prevention of an ERCP in these patients. Cholangitis was diagnosed in 92 patients, divided into 44.6% suspected and 55.4% definitive cases. Forty-six patients (50.0%) underwent additional imaging before ERCP despite the guideline recommending immediate ERCP in the presence of cholangitis. On the other hand, 11 patients with cholangitis did not undergo ERCP, given the absence of abnormalities on additional imaging (9 suspected and 2 definitive, respectively).

**Table:** **Table 3** Additional imaging and ERCP procedure.

	Total (N = 305)	Low (N = 51)	Intermediate (N = 122)	High (N = 132)
Additional imaging performed -%	195 (59.3)	28 (54.9)	102 (83.6)	65 (49.2)
MRCP	82 (42.1)	7 (25.0)	46 (45.1)	29 (44.6)
EUS	47 (24.1)	8 (28.6)	21 (20.6)	18 (27.7)
CT	24 (12.3)	2 (7.1)	13 (12.7)	9 (13.8)
MRCP + EUS	32 (16.4)	10 (35.7)	17 (16.7)	5 (7.7)
MRCP + CT	6 (3.1)	0 (0.0)	3 (2.9)	3 (4.6)
EUS + CT	2 (1.0)	0 (0.0)	1 (1.0)	1 (1.5)
MRCP + EUS + CT	2 (1.0)	1 (3.6)	1 (1.0)	0 (0.0)
Additional imaging results - %*				
No abnormalities	55 (28.5)	12 (42.9)	32 (31.4)	11 (17.5)
CBD stones and/or sludge	113 (58.5)	12 (42.9)	53 (52.0)	48 (76.2)
Other findings	25 (13.0)	4 (14.3)	17 (16.7)	4 (6.3)
ERCP performed - %	182 (59.7)	13 (25.5)	61 (50.0)	108 (81.8)
ERCP successful	161 (88.5)	13 (100)	56 (91.8)	92 (85.2)
ERCP findings† - %				
No abnormalities	21 (13.0)	4 (30.8)	11 (19.6)	6 (6.5)
Yes, CBD stones and/or sludge	136 (84.5)	8 (61.5)	43 (76.8)	85 (92.4)
Other findings	4 (2.5)	1 (7.7)	2 (3.6)	1 (1.1)
Intraprocedural complications ^‡^ - %	19 (10.5)	3 (23.1)	7 (11.7)	9 (8.3)
ERCP after same session EUS - %	44 (24.2)	7 (53.8)	21 (34.4)	16 (14.8)
*Two patients in the high-likelihood group underwent additional imaging without results. One EUS could not pass the esophagus due to a stricture and one MRCP failed due to claustrophobia. ^†^ ERCP findings are a percentage of successful ERCP. ^‡^ Intraprocedural complications percentage of ERCPs performed. CBD, common bile duct; CT, computed tomography; ERCP, endoscopic retrograde cholangiopancreatography; EUS, endoscopic ultrasound; MRCP, magnetic resonance cholangiopancreatography.				

### ERCP

As shown in Table 3 , ERCPs were performed most frequently in the high-likelihood group (81.8% vs 50.0% and 25.5% in the intermediate- and low-likelihood group, respectively). In total, 182 ERCPs were performed, of which 88.5% were successful. A trend was found toward higher procedure failure rates when ERCP was performed without prior imaging by EUS and/or MRCP compared with when additional imaging was performed (16.5% vs 7.8% unsuccessful ERCPs, respectively). The overall intraprocedural complication rate was 10.5%.

CBD stones were detected in 136 ERCP procedures (84.5%). In the low-likelihood group, relatively more ERCP procedures were performed with no abnormalities (30.8%), whereas in the high-likelihood group, no CBD stones were found in only 6.5% of the procedures.

Only 44 of 182 ERCPs (24.2%) were performed after an EUS in the same session, whereas 62 (34.1%) procedures were performed directly without any prior additional imaging. Four of 57 (7%) and two of five (40%) directly performed ERCPs in the high-likelihood group and intermediate-likelihood group respectively, showed no abnormalities.

### Follow-up

Median duration of hospital stay was 1 day (IQR 1–3) ( Table 4 ). PEP was the most common reason for prolongation of hospital stay. PEP occurred in 13 patients with an incidence of 7.1%. Therefore, PEP was responsible for more than half of the total of 25 complications. Number and severity of complications were comparable between likelihood groups.

**Table:** **Table 4** Follow-up.

	**Total (N = 305)**	**Low (N = 51)**	**Intermediate (N = 122)**	**High (N = 132)**
Length of stay – median (IQR)	1 (1–3) N = 180	1 (0–1) N = 17	1 (1–1) (N = 60)	2 (1–5) (N = 103)
Adverse events				
Post-ERCP pancreatitis	13	1	0	12
Cholangitis	6	0	3	3
Hemorrhage	5	0	3	2
Perforation	1	0	1	0
Other*	8	2	2	4
Adverse event severity ^†^				
Grade I	18	3	3	12
Grade II	7	0	2	5
Grade III	7	0	4	3
Grade IV	1	0	0	1
Grade V	0	0	0	0
*Other complications included pain (N = 6) and unexplained fever (N = 2). ^†^ According to Clavien-Dindo classification. ERCP, endoscopic retrograde cholangiopancreatography; IQR, interquartile range.				

## Discussion

ERCP is the gold standard in treatment for CBD obstructions, most frequently caused by gallstones 5 . A disadvantage of performing ERCP is the post-procedural complication risk of approximately 12% 11 . Therefore, the ESGE guideline for gallstone management stratifies patients into three groups based on likelihood of CBD stones, with direct ERCP only recommended in the high-likelihood group 5 . This multicenter observational study analyzed adherence to the ESGE guideline recommendations regarding stratification of patients with suspected choledocholithiasis in low-, intermediate-, and high-likelihood groups. Overall adherence was 59.7%, with the highest adherence observed in the intermediate-likelihood group for choledocholithiasis (83.6%) and the lowest in the high-likelihood group (43.2%). The low-likelihood group had an adherence rate of 45.1%.

Diagnostic uncertainty among physicians may contribute to an increase in additional imaging not indicated by the guideline. This caution could be driven by the high incidence of complications after ERCP. Conversely, overall guideline adherence across medical practice is only 77% 20 and in our study only 59.7%. Achieving 100% guideline adherence is not feasible due to patient preferences or situations in which adherence to the general recommendation is not desirable or possible for specific patients.

In comparison with other studies published after 2019, in which patients were also stratified according to the ESGE guideline, our study included a larger cohort of patients with a low-likelihood for cholelithiasis within a multicenter setting, achieving a higher level of generalizability 12 21 . Tunruttanakul et al. included only patients in a tertiary center, while EUS was not available during the study period. Sperna Weiland et al *.* included only patients who underwent ERCP, accounting for their low inclusion of low-likelihood patients 12 . There is a possibility that the low-likelihood patients were also undercounted in our study due to the inherent selection bias of using billing code data.

By conducting a complete diagnostic workup, 23 ERCPs and/or additional imaging procedures were avoided. Diagnostic tests and treatment strategies for those who had already undergone cholecystectomy revealed a high percentage of incomplete diagnostic assessments (26/77, 33.8%). Confirmation bias could have played a role in this difference, with physicians possibly relating a higher a priori chance of gallstone etiology to non-specific symptoms. Previous studies suggest that not each patient with suspicion of symptomatic cholelithiasis after cholecystectomy requires an ERCP, justifying the workup recommended by the guideline 22 23 .

Similarly, patients diagnosed with cholangitis caused by choledocholithiasis, who are categorized as high-likelihood for requiring ERCP, do not always need an ERCP because of the possibility of spontaneous passage of the CBD stone. The guideline does not specify if suspicion of cholangitis, according to the Tokyo guideline, 16 is enough to determine whether a patient has a high likelihood of choledocholithiasis, or if biliary dilatation must preferably be present on the first imaging modality (e.g. transabdominal US) 5 . Our findings illustrate that ERCP was no longer indicated in 11 of the 46 patients with a (suspicion of) cholangitis after undergoing additional imaging.

An argument for increased use of additional imaging is lower echogenicity in patients presenting with suspected cholelithiasis and being overweight or obese 24 25 . This phenomenon also leads to increased risk of having cholelithiasis 26 .Due to additional imaging, a reduction of 28.2% (55 of 195) of performed ERCPs was found, which was less than the 40.4% described in the study by Maruta et al 27 . It is worth noting that additional imaging was performed in an unselected (e.g. not stratified) population in their study. Furthermore, the Japanese population in their study is different than our predominantly White population, because incidence of choledocholithiasis is reported to be lower in Japan 1 28 . In our opinion, the benefit of preventing complications during and after ERCP makes it a worthwhile strategy to perform additional imaging prior to ERCP.

Another potential advantage of performing additional imaging prior to ERCP is the apparently lower ERCP failure rate in the group who underwent additional imaging. Because our study was not powered for this outcome, this needs to be confirmed in subsequent studies.

We observed a PEP rate of 7.1%. Based on a PEP risk of approximately 10%, as described in the meta-analysis by Akshintala et al *.* 6 , the 55 ERCP procedures that were prevented by additional imaging theoretically could have prevented five cases of PEP.

### Limitations

Unfortunately, due to the retrospective design of this study, missing data are unavoidable. Another limitation of the study is that EUS was only available in one of the two participating centers; therefore, proportionately more MRCPs were performed in our cohort. MRCPs, transabdominal US, and laparoscopic cholecystectomies could be performed in both hospitals. The proportionally higher number of MRCPs is comparable to the study by Jagtap et al 8 . Their study showed that MRCP and EUS exhibit comparable sensitivities. In our opinion, EUS is the preferred modality, owing to its capacity to be conducted concurrent with ERCP because the interval between additional imaging and ERCP procedure is minimized, which affects the likelihood of confirming presence of choledocholithiasis during ERCP 12 . Conducting an EUS in the same session also reduces risk of sedation-related complications 29 .

In an ideal setting, all endoscopists who perform ERCP are also skilled at performing EUS and this should be part of the training curriculum for advanced endoscopy training or fellowships 30 . If an interval between EUS and ERCP of several days is unavoidable, and the interval between MRCP and ERCP is shorter, MRCP would be the preferred additional imaging modality.

## Conclusions

Stratification according to the ESGE guideline is useful to reduce the number of unnecessary additional imaging studies or futile ERCPs. For high-likelihood patients, it is worthwhile to perform a same-session EUS prior to ERCP instead of directly performing an ERCP to decrease the number of negative ERCPs, consequently preventing ERCP-related complications.

## Supplementary Material

Supplementary Material Zusatzmaterial
