# Supplementary material for: Application of EUS or MRCP prior to ERCP in patients with suspected choledocholithiasis in clinical practice
Source: Endosc Int Open. 2025 Jan 7;13:a24750099. doi: 10.1055/a-2475-0099 (PMC11863547; doi:10.1055/a-2475-0099)
Supplement: Supplementary file 1 — Supplementary Material [file 10-1055-a-2475-0099_24804096.pdf]

Supplementary material

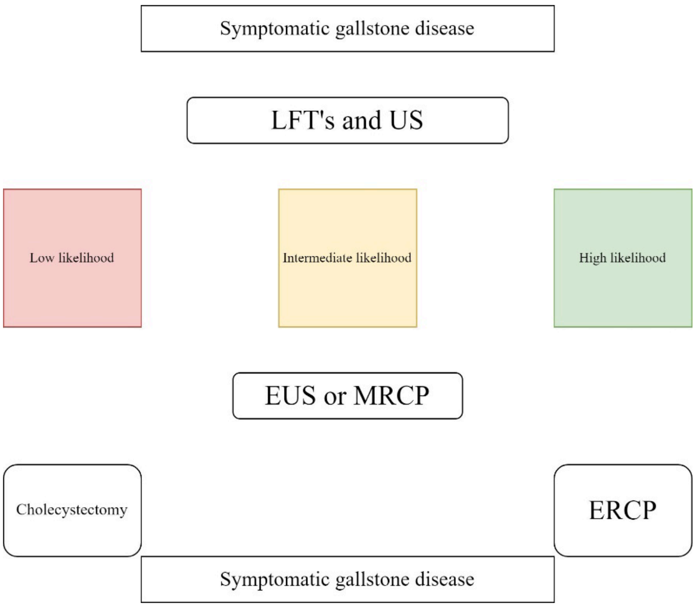

Supplemental figure 1: Flowchart of ESGE stratification as adapted from the guideline

Supplementary material

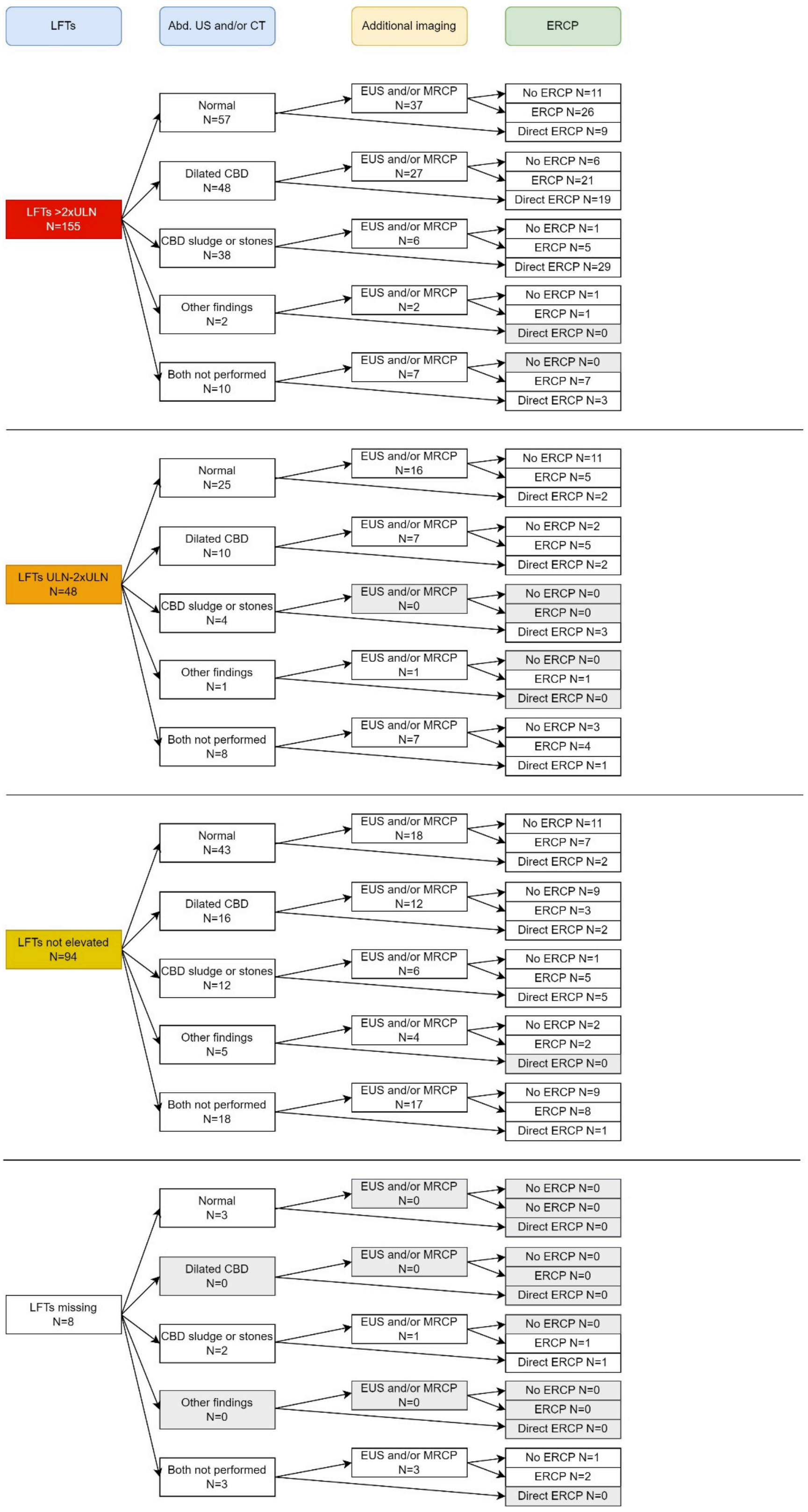

Supplemental figure 2: Management strategy stratified by LFT results
